# Deep Learning-Based Secure MIMO Communications with Imperfect CSI for Heterogeneous Networks

**DOI:** 10.3390/s20061730

**Published:** 2020-03-20

**Authors:** Dan Deng, Xingwang Li, Ming Zhao, Khaled M. Rabie, Rupak Kharel

**Affiliations:** 1 School of Information Engineering, Guangzhou Panyu Polytechnic, Guangzhou 511406, China; dengdan@ustc.edu; 2 School of Physics and Electronic Information Engineering, Henan Polytechnic University, Jiaozuo 454000, China; 3 CAS Key Laboratory of Wireless-Optical Communications, University of Science and Technology of China, Hefei 230027, China; zhaoming@ustc.edu.cn; 4 Department of Engineering, Manchester Metropolitan University, Manchester M15 6BH, UK; k.rabie@mmu.ac.uk; 5 Department of Computing and Mathematics, Manchester Metropolitan University, Manchester M15 6BH, UK; r.kharel@mmu.ac.uk

**Keywords:** physical-layer security, deep learning, imperfect CSI, heterogeneous networks, channel estimation

## Abstract

Perfect channel state information (CSI) is required in most of the classical physical-layer security techniques, while it is difficult to obtain the ideal CSI due to the time-varying wireless fading channel. Although imperfect CSI has a great impact on the security of MIMO communications, deep learning is becoming a promising solution to handle the negative effect of imperfect CSI. In this work, we propose two types of deep learning-based secure MIMO detectors for heterogeneous networks, where the macro base station (BS) chooses the null-space eigenvectors to prevent information leakage to the femto BS. Thus, the bit error rate of the associated user is adopted as the metric to evaluate the system performance. With the help of deep convolutional neural networks (CNNs), the macro BS obtains the refined version from the imperfect CSI. Simulation results are provided to validate the proposed algorithms. The impacts of system parameters, such as the correlation factor of imperfect CSI, the normalized doppler frequency, the number of antennas is investigated in different setup scenarios. The results show that considerable performance gains can be obtained from the deep learning-based detectors compared with the classical maximum likelihood algorithm.

## 1. Introduction

In recent years, the heterogeneous wireless network, which can support high-density and high-rate traffic, has attracted much research interest from both academic and industry sectors [1,2,3]. The cooperation between the macro base stations (BSs) and the femto BSs can greatly improve the quality of service (QoS) of the user equipment, as well as the spectrum efficiency and the energy efficiency [4]. Therefore, ultra-dense heterogeneous technology has been adopted as one of the potential solutions to the next-generation wireless sensor network [5].

On the other hand, the architecture of heterogeneous wireless networks brings up many challenges to the physical-layer security [6,7,8]. Several signal-level algorithms, such as beamforming [9,10], artificial noise [11,12] or stochastic geometry approach [13,14], have been proposed as the classical solutions to the secure multiple-input multiple-output (MIMO) communication scheme. Consequently, as an improvement, deep learning-based secure communication has been proposed to protect the message transmission to the legitimate users [15]. However, due to the noise and the signal distortion or delay of channel estimation, it is difficult to obtain the ideal channel state information (CSI) from both the legitimate users and the eavesdroppers in most of practical scenes [16,17,18,19,20,21]. For example, authors in [22,23,24] derived a new outage probability expression and optimize the transmission capacity under different assumptions.

Deep learning technology has already shown astonishing capabilities in dealing with complex network in mobile communications, such as channel estimation, signal detection, modulation recognition and channel equalization. For example, Ref. [25] used a 2D image to present the time-frequency channel fading matrix. By using a super-resolution network and a denoising network, the proposed scheme produces more accurate channel estimation. Under the constraint of one-bit quantization, authors in [26] proposed deep neural network-based auto-encoder for OFDM receiver. Modulation recognition algorithm was considered in [27], where a convolutional neural network followed by a long short-term memory as the classifier was adopted to improve the robustness for modulation recognition. A CNN-based channel prediction scheme was designed in [28] in massive MIMO systems under channel aging effects, where an autoregressive network was used to model the temporal channel correlation of the wireless channel. Considering perfect free space optical communications, a pilot independent deep learning-based channel estimator was proposed in [29,30]. Simulation results indicated that the proposed scheme provided close enough performance to the perfect channel estimation scheme.

Although there are some works on learning-based channel estimation algorithms, with imperfect CSI, the impacts of CNN-based MIMO detector on physical-layer security performance is still an open question. Motivated by that, in this paper, we will discuss the deep learning-based secure MIMO communication algorithm for heterogeneous networks with imperfect CSI. In the heterogeneous networks, there exist both macro BSs and femto BSs, which can serve a group of users within different areas. In general, the macro BS provides the massive connections and the large-scale cell coverage in the hot spots. The femto BS can help enlarge the wireless coverage and the data rates of the user in network edge. In some practical scenarios, secure messages transmitted from the macro BS to the users may be intercepted by the femto BS. In this case, with the help of zero-forcing algorithm [31], the macro BS chooses null-space eigenvectors to prevent information leakage to the femto BS. Classically, the receiver obtains the CSI through pilots signal transmitted from the base station, and there exists a time difference between the channel estimation and the data packet transmission. Thus, the estimated CSI is an imperfect version of the instant of packet transmission, and the zero-forcing secure MIMO algorithm should be re-designed. In this paper, two deep CNN-based detectors are proposed, and a training set with imperfect CSI as well as the original messages or ideal CSI is fed to the CNN model to produce the refined CSI. Simulation results show that the proposed deep learning-based detectors outperform the classical maximum likelihood detector (MLD), especially in small correlation factor cases. The impacts of system parameters, such as the correlation factor, the antenna number, are evaluated in different setup scenarios.

The main contributions of this paper are as follows:

- We employ the deep learning-based technique for secure MIMO communications in heterogeneous networks, which can exploit the benefits of CNN learning model to produce more accurate CSI and meanwhile reduce the bit error rate (BER) of the receiver.
- We provide the detailed framework of deep learning-based detectors, where imperfect CSI as well as the original messages or ideal CSI are included in the training set, and can be used in different application scenarios.
- We present simulation results for deep learning-based detectors in heterogeneous networks. With the help of the CNN technique, the proposed detectors show obvious performance gain over the MLD with acceptable computational cost.

*Notations:* We use $\mathrm{CN}(\mu ,\sigma^{2})$ to represent the circularly symmetric complex Gaussian random variable with mean μ and variance $\sigma^{2}$, $f_{X}\left(x\right)$ and $F_{X}\left(x\right)$ denote the probability density function (PDF) and cumulative distribution function (CDF) of a random variance *x*, respectively, diag(A) is a row vector consisting of all diagonal elements of A, $A^{*}$ is the conjugate transpose of the A, and $H_{FM}$ denotes the wireless channel fading matrix from *M* to *F*.

## 2. Related Work

Considering Gaussian wiretap channel, Fritschek et al. [32] introduced an auto-encoder to model the noised wireless channel with a novel security loss function. The generative model of the auto-encoder was trained to encode a message such that the eavesdropper cannot decode it correctly. Results show that the proposed scheme learns a trade-off between legitimate communication rate and secrecy capacity. With the help of the channel’s statistical characteristic in relay networks, the authors in [33] proposed a new deep learning-based algorithm to design secure beamforming vector. Considering visible light communication, Xiao et al. [34] proposed deep reinforcement learning (DRL)-based secure communications strategy. Since the optimization of the system secrecy rate is non-convex and NP-hard, a suboptimal solution on beamforming vector can be obtained by introducing zero-forcing beamforming gain to the eavesdropper.

With perfect CSI, the deep feedforward neural network (DFNN) with three layers was adopted for time-slot wireless powered system in [35]. In the proposed scheme, the tuple system parameters, such as the time allocation factor, the power allocation factor as well as the rate of the wiretap channel were produced by the DFNN. During the training phase, the output of the DFNN was compared with the optimal system parameters obtained from exhaust search, and the mean squared error (MSE) was adopted as the performance loss function. Numerical results of the DFNN and the optimal parameters were provided to validate the proposed scheme. Using Stackelberg equilibria, the authors in [36] proposed a secure mobile crowd-sensing (MCS) scheme, where the DRL technique was adopted to derive the optimal MCS policy.

## 3. System Model

**Figure 1:**
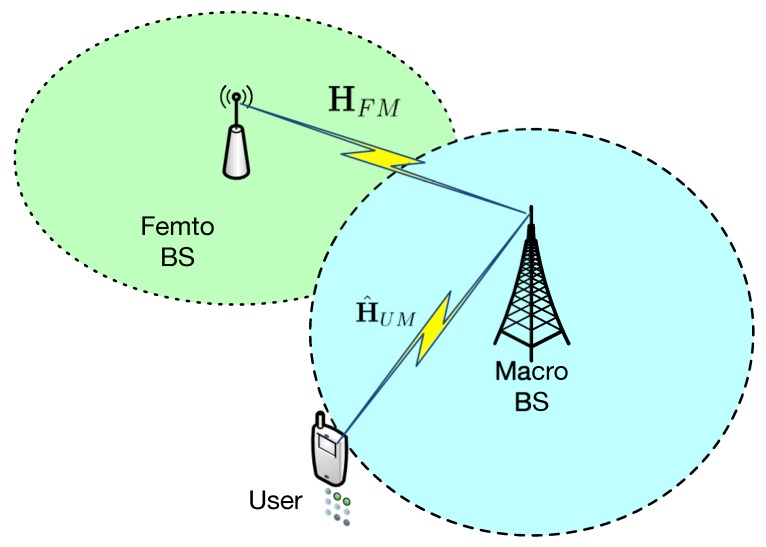
Secure MIMO communications for heterogeneous networks.

Figure 1 depicts the model of secure MIMO communications for heterogeneous networks. In the considered system, there is a macro BS, a femto BS and a terminal user. It is assumed that three types of nodes are all equipped with multiple antennas, and the numbers of antennas are denoted as $N_{M}$, $N_{F}$ and $N_{U}$, respectively. In the heterogeneous networks, macro BS and femto BS work cooperatively to provide the wireless coverage. Specifically, the users located in the hot spots area are associated with the macro BS, and the users located in the network edge are served by the femto BS. We use $H_{FM}$ and $H_{UM}$ to denote the wireless channel fading matrix from the macro BS to the femto BS and the users, respectively.

In addition, the channel fading matrix is modeled as time-varying and flat fading using the classical Jakes model [37]. Thus, the correlation coefficient between adjacent samples is given as

(1) $$\begin{array}{c} \rho =J_{0}\left(2\pi f_{d}\right), \end{array}$$

where $f_{d}$ is the normalized doppler frequency spread and $J_{0}\left(\cdot \right)$ is the zero-order Bessel function of the first kind.

Thus, the channel fading matrix can be calculated as

(2) $$\begin{array}{c} H_{UM}\left(n\right)=\rho H_{UM}\left(n-1\right)+\sqrt{1-\rho^{2}}N_{UM}\left(n\right), \end{array}$$

where *n* denotes the sample time and $N_{UM}\left(n\right)$ denotes the additional white noise matrix with the same size of $H_{UM}\left(n\right)$. The same equation can be applied on $H_{FM}$ as follows:

(3) $$\begin{array}{c} H_{FM}\left(n\right)=\rho H_{FM}\left(n-1\right)+\sqrt{1-\rho^{2}}N_{FM}\left(n\right). \end{array}$$

Please note that in the following sections, the sample time *n* is omitted without loss of generality.

It is well-known that the deep learning networks can effectively capture the correlation features of the training data set. Spatial correlation or antenna correlation, which may achieve dimensionality reduction, is an important challenge for MIMO systems [38,39]. Also, it will be a direction of our future work.

Since the time correlated MIMO channel model is adopted in this paper, we can use DCNN to obtain more accurate CSI from the outdated CSI. Actually, considering the phase rotation introduced by the channel matrix, the outdated CSI is necessary to assist the data recovery at the user.

Due to the open nature of the heterogeneous networks, the femto BS may intercept the signal transmitted from the macro BS to the users. To prevent the information leakage to the femto BS, the macro BS can zero-forcing the equivalent channel matrix of the femto BS by the null-space technique. In this case, the macro BS first obtains the CSI $H_{FM}$ between the macro BS and the femto BS. Then the null-space eigenvectors can be produced by applying eigenvalue decomposition on the autocorrelation matrix, i.e., $H_{FM}^{*}H_{FM}$, that is

(4) $$\begin{array}{c} \left(v,V\right)=Eig\left(H_{FM}^{*}H_{FM}\right), \end{array}$$

where Eig(·) denotes the eigenvalue decomposition, v denotes the eigenvalues in ascending order and V are the corresponding eigenvectors. Considering the size limitation for both the femto BS and users, it is reasonable to assume that the antenna number of the macro BS is larger than the femto BS and users, i.e., $N_{M}>N_{F},N_{M}>N_{U}$. Thus, the number of zero eigenvalues can be given as

(5) $$\begin{array}{c} N_{D}=N_{M}-N_{F}. \end{array}$$

Please note that $N_{D}$ is also the number of null-space vectors for $H_{FM}$, which is given as

(6) $$\begin{array}{c} B=V_{:N_{D}} \end{array}$$

where $V_{:N_{D}}$ denotes the first $N_{D}$ column vectors of V. Thus, the beamforming matrix B, which is used by the macro BS to transmit messages to its associated user, lies in the null-space of $H_{FM}$. Then the signal received at terminal user can be expressed as

(7) $$\begin{array}{c} y=\sqrt{P}H_{UM}Bx+N, \end{array}$$

where *P* is the transmission power of the macro BS, x∈CN(0,I) is the original message transmitted from macro BS with size $N_{D}$ and $N\in \mathrm{CN}(0,\sigma^{2}I)$.

Since the equivalent channel fading matrix of the femto BS is zero-forced, we only need to observe the signal-noise-ratio (SNR) of the associated user. We define the average normalized SNR received at the user as

(8) $$\begin{array}{c} \gamma =\frac{P}{\sigma^{2}N_{M}}. \end{array}$$

Classically, the receiver obtains the wireless CSI through pilot signals transmitted from the BS, and there exists a time difference between the channel estimation and the data packet transmission. Thus, the estimated CSI is an imperfect version of the instant of packet transmission. To reduce the analysis complexity, it is assumed that the estimation of $H_{FM}$, while that of $H_{UM}$ is imperfect. Specifically, the imperfect equation is modeled as

(9) $$\begin{array}{c} {\hat{H}}_{UM}=\sqrt{\xi }H_{UM}+\sqrt{1-\xi }N_{UM}, \end{array}$$

where ξ is the correlation factor of the imperfect version of channel matrix.

The standard maximum likelihood detector (MLD) with imperfect CSI can be employed to detect the original message as

(10) $$\begin{array}{c} \hat{x}=\arg\min_{x\in \Omega }{∥y-{\hat{H}}_{UM}x∥}^{2}, \end{array}$$

where Ω is the all possible constellations set.

## 4. Deep CNN-Based Detector

The imperfect CSI, which is introduced by the noise or delay of channel estimation, will greatly deteriorate the system performance of standard MLD. To overcome the effects of the imperfect CSI in secure MIMO communications, two types of deep CNN (DCNN)-based detectors are proposed in this section, which can be used in different application scenarios. The deep CNN models are first trained with predefined loss functions and then used to generate the refined CSI ${\tilde{H}}_{UM}$, which can be fed to the MLD to obtain the original message, i.e.

(11) $$\begin{array}{c} {\hat{x}}_{cnn}=\arg\min_{x\in \Omega }{∥y-{\tilde{H}}_{UM}x∥}^{2}. \end{array}$$

**Figure 2:**
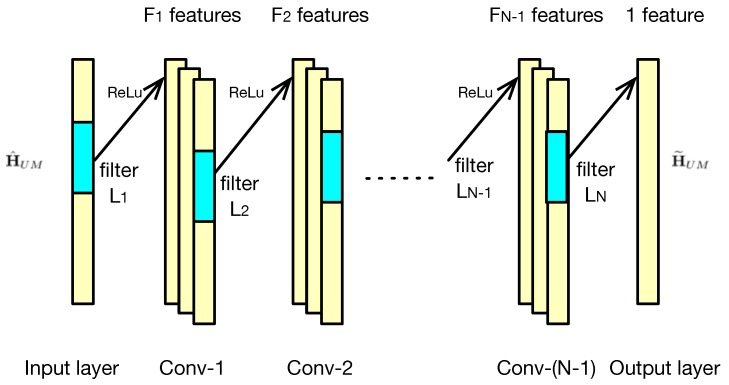
Details of DCNN model.

The details of the DCNN model is given as Figure 2, where there exist *N* one-dimension convolutional layers excluding the input layer. In the input layer, the channel fading matrix ${\hat{H}}_{UM}$ is reshaped as a column vector. Since only real data can be processed in the CNN model, the complex data of ${\hat{H}}_{UM}$ can be treated as two real channels [40]. Please note that each convolutional layer is followed by a ReLU activation function except the output layer. Moreover, in the *n*-th convolutional layer, there are $\left\{F_{n},n\in \left[1,N\right]\right\}$ features maps with filter length $\left\{L_{n},n\in \left[1,N\right]\right\}$. Specifically, in the output layer, there is only one feature map.

As to the detailed architecture of the DCNN, we must find the trade-off between complexity and performance. It is noted that the fully connected DNN, which may hold better performance, while its computational complexity is proportional to the square of the number of nodes. On the other hand, both the training data set and the training time required by fully connected DNN the is too large to be satisfied. There also exist some powerful CNN models, such as VGG [41] and ResNet [42], which improve the detection probability by increasing the depth of the models to 19 and 34, respectively. Specifically, the number of parameters for VGG-19 is up to 144M. Thus, to decrease the computational complexity, we must simplify the classical CNN models as follows. The architecture of DCNN model includes *N*=4 layers, and is described by $F_{n}$ and $L_{n}$ as $F_{n}=\left\{32,16,8,1\right\},L_{n}=\left\{36,3,3,36\right\}$.

In the following sections, two different methods, denoted as DCNN type-I and type-II, are introduced to train the DCNN model.

### 4.1. DCNN Type-I: Training with Accurate CSI

**Figure 3:**
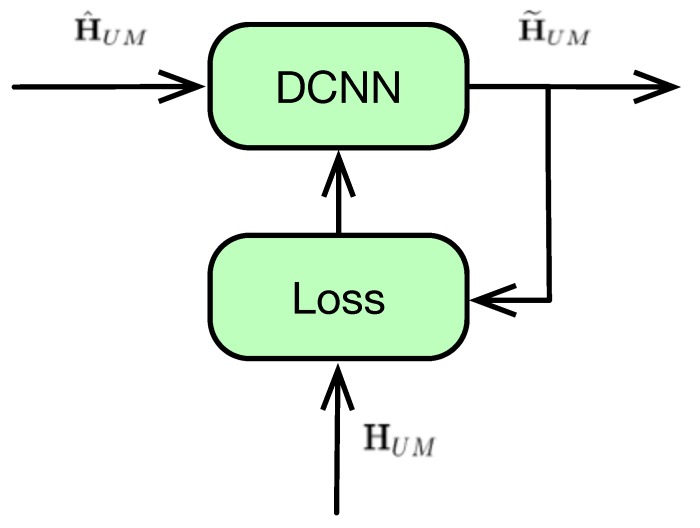
DCNN type-I: Training model of CNN with accurate CSI.

Figure 3 shows the first training method of DCNN-based detector with accurate CSI, which is denoted as DCNN type-I. In the training phase, a data set including both the imperfect CSI ${\hat{H}}_{UM}$ and the accurate CSI $H_{UM}$ is fed into the learning model. The loss function is defined as the mean square error between the output of the model ${\tilde{H}}_{UM}$ and the accurate CSI $H_{UM}$, i.e.

(12) $$\begin{array}{c} \epsilon_{I}=\frac{∥{\tilde{H}}_{UM}-H_{UM}{∥}^{2}}{N_{U}N_{M}}. \end{array}$$

During the DCNN model training, the loss function is calculated batch by batch and used to optimize the weight and the bias of the DCNN model [43]. Please note that the accurate CSI is necessary for DCNN type-I, which is used to calculate the model loss function. Thus, the application of DCNN type-I is limited, because in some practical scenarios, it is difficult to obtain the accurate CSI especially in the wireless MIMO communications. Therefore, another type of DCNN is proposed to overcome this limitation.

### 4.2. DCNN Type-II: Training with Original Message

**Figure 4:**
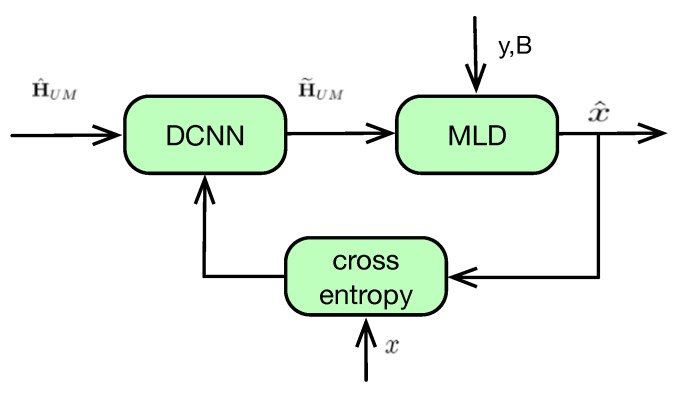
DCNN type-II: Training model of CNN without accurate CSI.

The training architecture of DCNN type-II is given as in Figure 4, where accurate CSI is not needed. Instead, the output of DCNN ${\tilde{H}}_{UM}$ is used in MLD and obtain the likelihood of each candidate message as follows:

(13) $$\begin{array}{c} {\hat{q}}_{i}=\exp\{-∥y-{\tilde{H}}_{UM}Bx_{i}{∥}^{2}\},x_{i}\in \Omega . \end{array}$$

By using of the SoftMax function, the normalized likelihood probability of each candidate message can be given as

(14) $$\begin{array}{c} q_{i}=\frac{{\hat{q}}_{i}}{\sum_{i=1}^{\left|\Omega \right|}{\hat{q}}_{i}}. \end{array}$$

We use $p_{i},i\in \left[1,|\Omega |\right]$ to denote the correct probability of each candidate message. That is $p_{i}=1$ if *i*-th candidate message is correct, otherwise $p_{i}=0$. Inspired by the information theory, the cross-entropy can be used to quantify the difference between two probability vectors.

Accordingly, for probability distributions of $q_{i}$ and $p_{i}$, we can calculate the cross-entropy as follows:

(15) $$\begin{array}{c} C\left(p,q\right)=\sum_{i=1}^{\left|\Omega \right|}p_{i}log_{2}\frac{1}{q_{i}}. \end{array}$$

Then, the cross-entropy C(p,q) can be used as the loss function to train the DCNN model.

Compared with the loss function in (12), only the received signal *y*, the beamforming matrix B and the original message *x* are needed, which enlarge the application scenarios of the DCNN type-II. On the other hand, without the help of the accurate CSI, DCNN type-II leads to deteriorated performance compared with type-I, which can be validated in the simulation results.

Please note that the DCNN model could also output the ground-true symbol *x* directly in a supervised manner, and the outdated MIMO channel matrix could be further employed as side information by inputting it to the DCNN. We use DCNN type-III to denote the new DCNN model. Although the detailed architectures of DCNN type-II and the suggested DCNN type-III are different, they are functionally equivalent as a black box with DCNN kernels. In other words, the MLD module in DCNN type-II can be seen as part of the functions of DCNN type-III.

## 5. Simulation Results

In this section, simulation results are provided to verify the proposed DCNN models. The impacts of system parameters, such as the correlation factor of imperfect CSI ξ, the normalized doppler frequency $f_{d}$, the number of antennas is evaluated in different setup scenarios. Since the equivalent channel fading matrix of femto BS is zero-forced, BER of users with different detectors is used to evaluate the system performance.

Specifically, QPSK modulation is employed in all simulation setups. Since the constellations of QPSK modulation is 2-D complex signal, we can generalize the setup to an arbitrary modulation order. We set the data packet length as 600 bits, and each batch consists 10 data packets. During the training phase, a training data set with 10000 batches as well as a validation data set with 1000 batches are fed to the DCNN, and a test data set with 1000 batches is used to evaluate the BER performance of the proposed schemes. The popular TensorFlow framework [44] is adopted in our simulations, while the adaptive moment estimation (Adam) optimizer is used to minimize the loss value during training phase. In particular, the optimization parameters are listed as follows: learning rate is 0.001, $\beta_{1}=0.9$, $\beta_{2}=0.999$, $\epsilon =10^{-8}$. Since the testbed of our paper is under construction at this moment, we will present experiment results on real datasets in future works.

**Figure 5:**
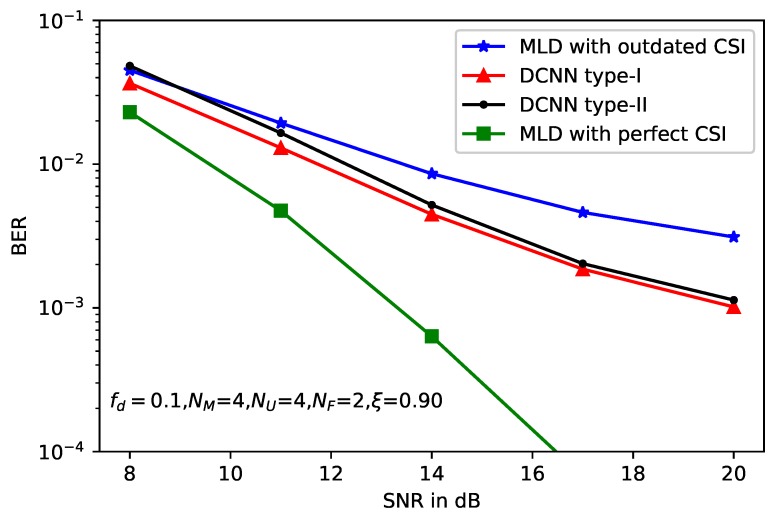
BER performance of deep learning-based MLD with ξ=0.9.

Figure 5 depicts the BER performance versus the SNR of the system with MIMO setup as $N_{M}=4,N_{U}=4,N_{F}=2$, the normalized doppler frequency $f_{d}=0.1$, and the correlation factor of imperfect CSI ξ=0.90. The BER of three types of detectors, such as standard MLD, DCNN type-I and DCNN type-II are compared. As a benchmark, the BER curves obtained by MLD with the perfect CSI is also presented. We can see from this figure that the outdated CSI has obvious adverse effects on BER performance. As shown in this figure, in the high SNR region, DCNN-based detectors show a performance gain of about 4dB in comparison to the standard MLD. The reason is that the former can refine the imperfect channel matrix and produce more accurate CSI, then the BER of the system can be improved.

**Figure 6:**
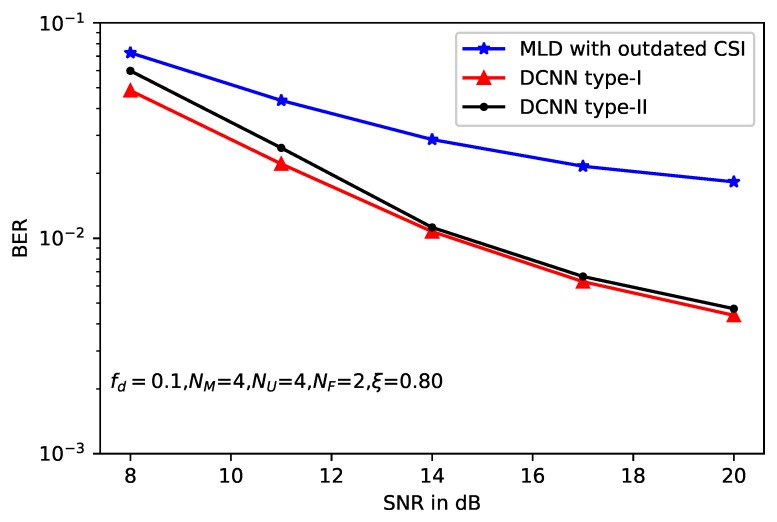
BER performance of deep learning-based MLD with ξ=0.8.

**Figure 7:**
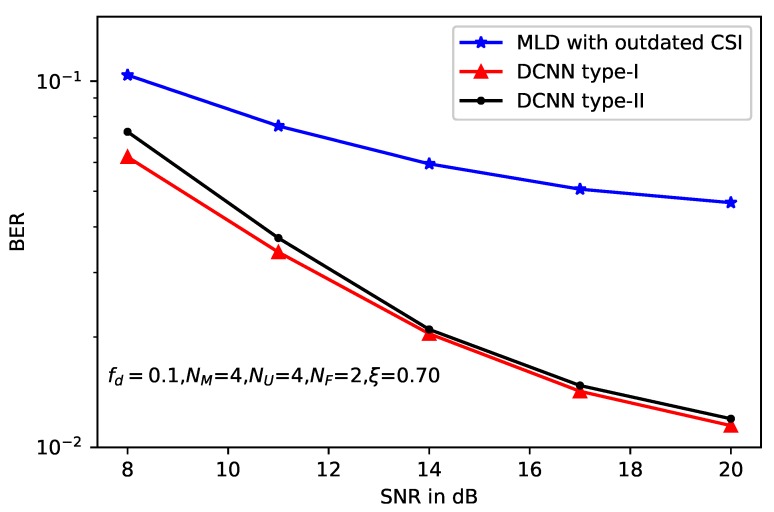
BER performance of deep learning-based MLD with ξ=0.7

Moreover, two types of DCNN-based detectors show almost the same performance with slight gap. Similar results can be obtained from Figure 6 and Figure 7, where the correlation factors of imperfect CSI are ξ=0.8 and ξ=0.7, respectively. However, accurate CSI is necessary for DCNN type-I, which is used to calculate the model loss function. Thus, the application of DCNN type-I is limited, because in some practical scenarios, it is difficult to obtain the accurate CSI especially in the wireless MIMO communications. Compared with the loss function, only the received signal *y*, the beamforming matrix B and the original message *x* are needed, which enlarge the application scenarios of the DCNN type-II. On the other hand, without the help of the accurate CSI, DCNN type-II leads to deteriorated performance compared with type-I.

**Figure 8:**
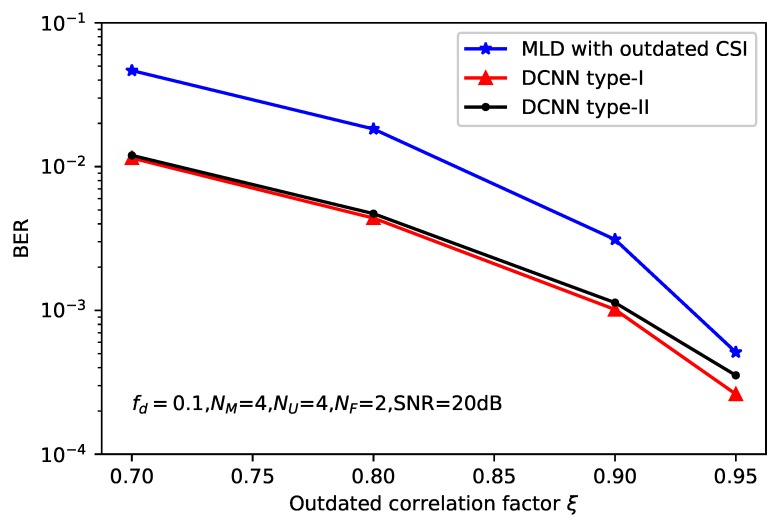
BER performance of deep learning-based MLD with SNR = 20 dB.

Figure 8 depicts the BER performance versus the correlation factor of imperfect CSI ξ, where the average SNR of the system is SNR = 20 dB. The BER of three types of detectors, such as standard MLD, DCNN type-I and DCNN type-II are compared. As shown in this figure, DCNN-based detectors indicate considerable performance gains relative to the standard MLD. This is because DCNN-based detectors can refine the imperfect channel matrix and produce more accurate CSI, hence the BER of the system can be improved. Moreover, two types of DCNN-based detectors show almost the same performance.

**Figure 9:**
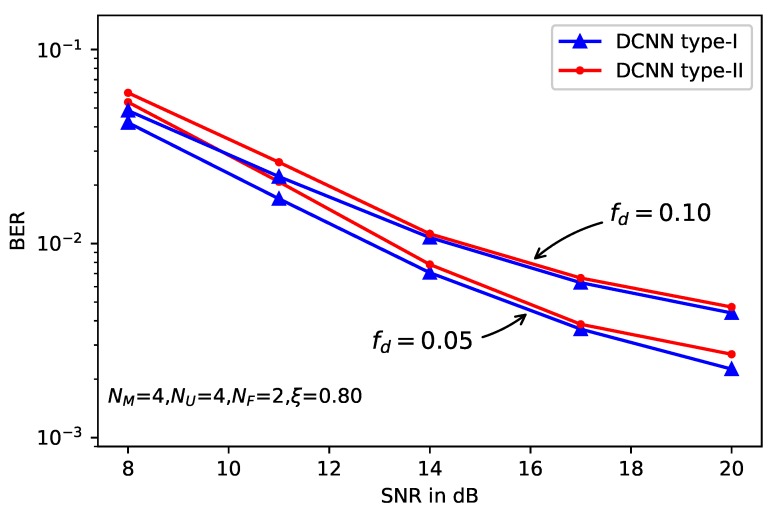
BER performance of deep learning-based MLD with different $f_{d}$.

The effect of the normalized frequency $f_{d}$ is present in Figure 9, where the MIMO configuration remains the same with previous setup. The BER curves of both DCNN training models are provided with $f_{d}=0.1$ and $f_{d}=0.05$, respectively. We can see from this figure that smaller $f_{d}$ produces better performance with a gain of about 4dB. The reason is that if $f_{d}$ is smaller, the channel fading matrix changes more slowly, and the wireless channel can be learned more efficiently by the DCNN. As a result, more accurate CSI can be produced and enhancing the BER performance.

**Figure 10:**
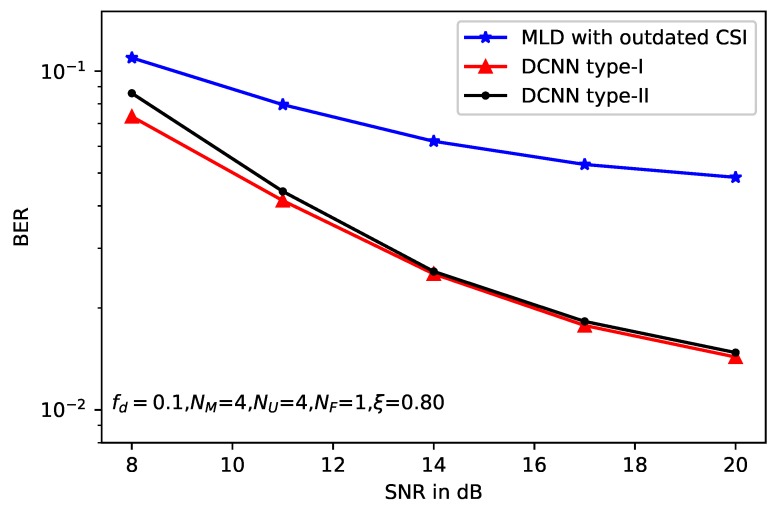
BER performance of deep learning-based MLD with $N_{F}=1$.

**Figure 11:**
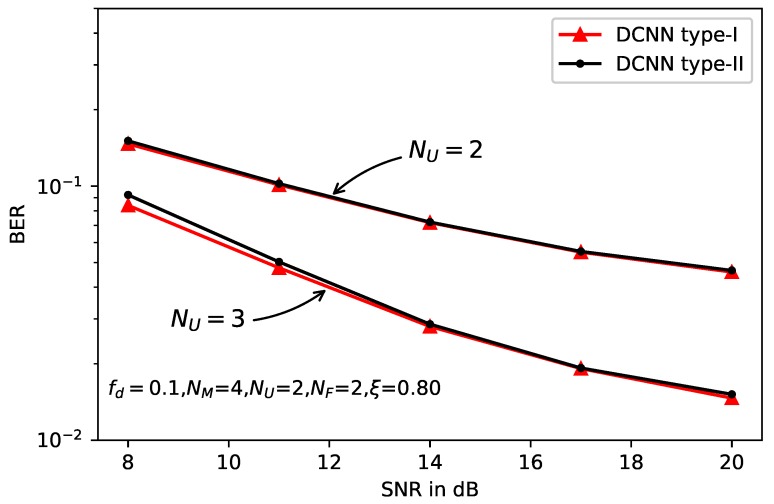
BER performance of deep learning-based MLD with different $N_{U}$.

Figure 10 and Figure 11 depict the effects of the antenna number on BER performance with the normalized doppler frequency $f_{d}=0.1$, and the correlation factor of imperfect CSI ξ=0.80. Specifically, the antenna configuration in Figure 10 is $N_{M}=4,N_{U}=4,N_{F}=1$, and the number of data stream $N_{D}=3$. In other words, the spectrum efficiency is higher than the previous setup. In Figure 11, the antenna configuration is $N_{U}=2$ and $N_{U}=3$, respectively. We can see from the two figures that the performance gain of DCNN with larger antenna number of user is obvious compared with the smaller antenna number, especially in the higher SNR region. Specifically, with $N_{F}=1$, the performance gain of DCNN to the standard MLD is about 6dB. The performance gain of $N_{U}=3$ is about 8dB than that of $N_{U}=2$. That reason is that the larger receiver antennas’ number can introduce more freedom of space diversity, thus the BER will be decreased greatly.

## 6. Conclusions

In this paper, we investigate two types of deep learning-based secure MIMO detectors for heterogeneous networks. In the considered system, the equivalent channel fading matrix of the femto BS is zero-forced through null-space eigenvectors. The BER of the associated user is adopted as the metric to evaluate the system performance. with the help of deep convolutional neural networks, the macro BS produces more accurate CSI. The impacts of system parameters, such as the correlation factor of imperfect CSI, the normalized doppler frequency, the number of antennas are investigated in different setup scenarios. Considerable performance gains can be obtained from the deep learning-based detectors compared with the classical maximum likelihood algorithm.
